# Microbial Keratitis in Kingdom of Bahrain: Clinical and Microbiology Study

**DOI:** 10.4103/0974-9233.48855

**Published:** 2009

**Authors:** Nada Al-Yousuf

**Affiliations:** From the Department of Ophthalmology, Salmaniya Medical Complex, Kingdom of Bahrain

**Keywords:** Keratitis, Corneal Ulcer, Pseudomonas Aeroginosa, Contact Lenses

## Abstract

**Background:**

Microbial keratitis is a potentially vision threatening condition worldwide. Knowing the predisposing factors and etiologic microorganism can help control and prevent this problem. This is the first study of its kind in Kingdom of Bahrain.

**Objective:**

To study the profile of microbial keratitis in Bahrain with special focus on risk factors, clinical outcome and microbilogical results.

**Methods:**

A retrospective analysis of all patients admitted in Salmaniya Medical Complex over a period of three years from January 2005 to January 2007 was performed. A total of 285 patients with keratitis were analysed. Non infectious corneal ulceration were excluded. Data collected from medical records were demographic features, predisposing factors, history of corneal trauma, associated ocular conditions, visual acuity at the time of presentation and the clinical course. Predisposing risk factors measured were contact lens use, presence of blepharitis, diabetes, lid abnormalities, dry eyes, keratoplasty and refractive surgery. For contact lens wearers any contact lens related risk factors that can lead to keratitis were measured. Pearson's chi-square test was used to carry out statistical analysis wherever required.

**Results:**

Contact lens wear, as a risk factor for microbial keratitis, formed 40% of the total study population. Other risk factors identified were dry eyes 24 cases (8%), 10 blepharitis (3%), 22 trauma (8%), abnormal lid position 14 cases (5%). 6 patients keratitis in a graft (2%), 3 had refractive surgery (1%). The most common causative organism isolated was pseudomonas aeroginosa (54%) followed by streptococcus 12%, staph 10%, other organisms 6%. 95% of contact lens wearers had pseudomonas Aeroginosa. This was statistically significant (p< 0.0001). The vast majority, 92% healed with scarring. 1% needed therapeutic keratoplasty and 7% lost to follow up. Risk factors in contact lens wearers were; 41 patients (36%) slept with the contact lenses. 12 (8%) had contact lens related trauma and 8 (7%) had poor hygiene. Sleeping with the contact lenses was statistically significant (p< 0.0001).

**Conclusion & Recommendation:**

Contact lens wear is the major risk factor for microbial keratitis in Bahrain. Pseudomonas aeroginosa was the commonest bacteria isolated. Sleeping with the contact lenses is the major risk factor among contact lens wearers. Majority of keratitis patients resulted in permanent scarring on the cornea. Educating the public, especially on contact lens care and precaution, can help reduce this visual morbidity.

Microbial keratitis is a potentially vision threatening condition that requires prompt diagnosis and treatment to prevent devastating outcomes. It may be caused by bacteria, fungi, viruses or parasites. It rarely occurs in the normal eye because of the cornea's natural resistance to infection. However, predisposing factors such as trauma, contact lens wear, dry eyes, ocular surface disorders and immunosuppression may alter the defence mechanism of the outer eye, and permit bacteria to invade the cornea. The incidence of this condition varies from 11 per 100 000 in the United States^1^ to 299 per 100 000 in developing countires.^2^ Microbial keratitis is a significant health problem. As can be expected, there are regional differences in the incidence, predisposing factors and microbiology profiles.

On a global level, predisposing risk factors for microbial keratitis vary tremendously with geographical location. Although trauma to the eye accounted for 48 to 65 % of all corneal ulcers in developing countries,^2^ at a large trauma referral centre in the United States, trauma accounted only for 27% of all cases.^3^ The major risk factor in the United States is contact lens wear which was reported to account for 52% of keratitis cases.^1^

Keratitis blunts the quality of life. Many patients are young, working adults who develop an unexpected infection from contact lenses or other injury. Considering the importance of this problem as a serious cause of visual loss, studies worldwide have reported the prevalence of microbial pathogens in their community, and identified the risk factors predisposing their population to this infection. This study was not done before in the Kingdom of Bahrain. Hence the understanding of the epidemiological features, risk factors and aetiological agents that occur in Bahrain, are important in rapid recognition, timely institution of therapy, optimal management and prevention of this disease entity. The purpose of this study is to determine risk factors related to microbial keratitis, to measure any contact lens malpractice in those with contact lens keratitis, and to identify pathogens causing keratitis at a tertiary referral centre in Kingdom of Bahrain.

## MATERIALS AND METHODS

### Patients

A retrospective analysis of all patients seen in Salmaniya Medical Complex over a period of three years from January 2004 to January 2006 was performed. A total of 285 patients with keratitis were analysed. All patients with infectious keratitis were included in the study. Corneal ulceration was defined as loss of corneal epithelium with underlying stromal infiltrate associated with signs of inflammation with or without hypopyon. Non infectious corneal ulceration such as Mooren's ulcer, sterile neurotropic ulcers, marginal keratitis and ulcers associated with autoimmune disorders were excluded. Data collected from medical records were demographic features, predisposing factors, history of corneal trauma, associated ocular conditions, systemic diseases, therapy received prior to presentation, visual acuity at the time of presentation and the clinical course. Predisposing risk factors measured were contact lens use, presence of blepharitis, history of trauma, diabetes, lid abnormalities such as entropion and trichiasis, presence of dry eyes, coexisting keratoplasty and history of refractive surgery. For contact lens wearers there was a section to indicate any malpractice that can lead to keratitis; sleeping with the contact lenses, irregular hygiene, trauma while inserting or removing the lenses, and use of home made saline solution.

### Clinical Examination and Laboratory Investigation

All patients underwent thorough slit-lamp biomicroscopic examination by an ophthalmologist. Clinical features were noted and a drawing made. After a detailed ocular examination, corneal scrapings were taken, using a sterile 21 gauge needle or a blunt Kimura spatula, from the leading edge and the bed of the ulcer by an ophthalmologist. The procedure performed under the magnification of slit-lamp after instillation of topical anaesthetic (Amethocaine or Benoxinate). The material obtained was spread onto labelled slides for Gram and Giemsa stains. The material scraped was also inoculated onto the surfaces of agar plates for bacterial and fungal growth. All patients received fortified Cephalosporin (Zinacef) and fortified aminoglycoside (Tobramycin) in addition to a flouroquinolone (Oflox).^4^

Pearson's chi-square test was used to carry out statistical analysis wherever required.

## RESULTS

### Demographic Study

Out of 285, 173 (61%) were females and 112 (39%) were males. 239 were Bahrainis (84%) and 46 (16%) were non Bahrainis. The mean age was 51. The mean age among contact lens wearers was 21.

### Microbial Keratitis Risk Factors

**Figure 1:**
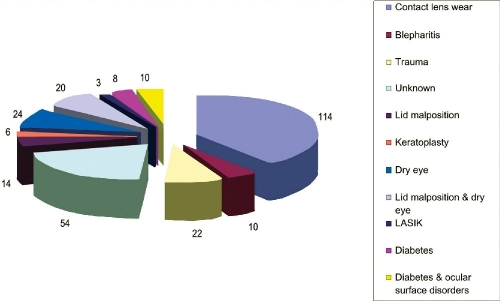
Risk factors related to Microbial keratitis in Kingdom of Bahrain

As shown in Fig. 1 contact lens wear is the major risk factor in this study. 114 cases (40%) were contact lens users. 24 cases (8%) had dry eyes, 10 had blepharitis (3%), 22 had trauma (8%) abnormal lid position 14 cases (5%). 6 patients had keratitis in a graft (2%), 8 patients were known diabetics and had no other risk factor (3%) and 3 patients had undergone LASIK (1%). Those who had combination of more than one risk factors were 38 (11%). Lid malposition and dry eyes were 20 cases (7%) and diabetics with either lid malposition or dry eyes or combination (ocular surface disorder) were 10 patients (4%). 54 patients forming 19% of all keratitis cases had no risk factors recorded.

### Microbiology Profile

**Figure 2:**
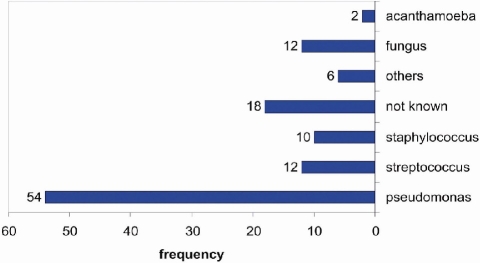
Type of microorganism isolated from corneal ulcers in patients with microbial keratitis

111 cases forming 39 % of all cases had positive culture yield for bacteria growth. The most common causative organism isolated was pseudomonas aeroginosa. It accounted for 54% of all cases. This is followed by streptococcus 12%, staph 10%, other organisms 6%. Among the other organisms were Moraxella, Bacillus, Corynebacterium, Serratia, and Haemophilus influenza. 95% of contact lens wearers with positive culture yield on microbiology study have grown pseudomonas Aeroginosa. This was statistically significant (p< 0.0001) 12 (4%) cases had fungal keratitis and 2 (0.7%) had acanthamoeba keratitis (Fig. 2).

### Clinical Course

#### Visual Acuity at Presentation

**Figure 3:**
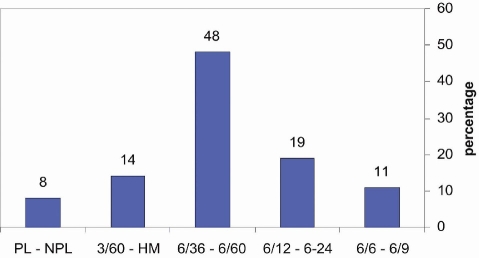
Visual acuity at presentation in patients with microbial keratitis

48% had vision between 6/36 and 6/60, 19% had vision between 6/12 and 6/24, 14% had visual acuity 3/60 – HM, 11% had 6/6 -6/9 vision and 8% had PL – NPL vision (Fig. 3).

The vast majority, 92% healed with scarring. 1% needed therapeutic keratoplasty and 7% lost to follow up (Table 1).

**Table 1:** Clinical Course of Microbial Keratitis

Clinical Outcome	Frequency
Healed with scar	262 (92%)

Needed keratoplasty	3 (1%)

Lost to follow up	20 (7%)

### Contact Lens Related Risk Factors

**Figure 4:**
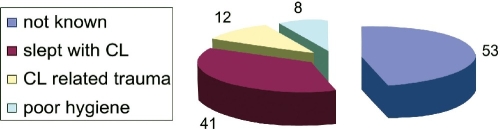
Risk factors associated with contact lens use

Out of the 114 contact lens wearers 53 patients (46%) either denied any contact lens related malpractice, or had no recorded information on misuse. 41 patients (36%) slept with the contact lenses. 12 (8%) had contact lens related trauma and 8 (7%) had combination with poor storage case hygiene, and irregular disinfection. None had used home made saline solution (Fig. 4).

Those with known risk factors related to contact lens wear. Sleeping with the contact lenses (36%), compared to other recorded risk factors (15%), the difference was statistically significant (p<0.0001).

## DISCUSSION

This is the first study of its kind in Kingdom of Bahrain. Contact lens wear is known to be a major risk factor for microbial keratitis.^5^,^6^ It was the leading risk factor in this study. This is consistent with other studies. Schaefer et al reported that contact lens wear was by far the predominant predisposing factor for bacterial keratitis.^7^ Similarly, in academic referral institutions in the United states, there was a well documented upward trend in the incidence of contact lens related ulcers.^8^,^9^ According to Hsiao and associates contact lens wear was also a major risk factor for microbial keratitis in their review of paediatric hospital cases with microbial keratitis.^10^

Bialasiewicz and co-workers who studied risk factors for microbial keratitis in Oman found that although contact lens wear was one of the commonest predisposing factors for keratitis, trachoma was the leading risk factor in their series. Moreover, they studied the effect of using traditional medicine on keatitis outcome. Traditional medicine was not represented in our study as is it not widely used in the Bahraini community. Furthermore, that the majority of our patients were contact lens users. Contact lens wearers mostly belong to younger age group in which traditional medicine is not practised.^11^ In Kingdom of Saudi Arabia, although the most important risk factor for keratitis was penetrating keratoplasty, they demonstrated an increase in contact lens wear as a predisposing factor.^12^ Bharathi and associates their epidemiologic study of microbial keratitis in South of India have shown that ocular surface disease was the commonest risk factor for microbial keratitis.^13^ Basak and co-workers from India have demonstrated that trauma was the major risk factor.^14^ In Honk Kong, Lam and colleagues have found that previous ocular disease and trauma were the main risk factors for microbial keratitis.^15^

In this study, refractive surgery was not one of the major risk factors for microbial keratitis it represented only 1% of all keratitis patients. Lam and coworkers from Honk Kong have found the same.^15^ The author believes that refractive surgery is an emerging risk factor for microbial keratitis. As refractive surgery expands further, there might be less contact lens related keratitis, and possibly more refractive surgery related microbial keratitis.^16^

Although contact lens wear was by far the leading cause of microbial keratitis in the present study, there is a striking low incidence of acanthamoeba keratitis as compared with Europe and North America.^17^,^18^ This is probably because of the paucity of fresh water in Bahrain. Moreover, the use of desalinated sea water to provide domestic water supplies may reduce the risk of acanthamoeba infection. In addition, the relatively smaller use of hot tubs, and the absence of swimming in fresh water, compared to residents of western countries might contributed to this low incidence.^19^,^20^ This was also demonstrated by Al-Mezaine and co-workers who have demonstrated low incidence of acanthamoeba keratitis in Saudi Arabia compared to Europe and North America.^21^

In this study 61% of cases culture were negative and therefore had no definite laboratory diagnosis. Possible reasons for negative cultures; some patients were already on topical medications when they arrive, defective scraping techniques by residents on call, and/or problems in microbiology transport and handling. In a study done in India, 80% of cases had culture negative.^22^ In Ghana, microscopy and culture results were negative in 60% of cases.^22^ In Honk Kong, 65% of patients had no positive culture yield. None of those studies have indicated their possible causes of the high negative culture rate.^15^ In Switzerland researchers have shown that in 86% of patients, bacteria were identified. In their study they mentioned that the patients who were culture negative, treatment with antibiotic had been already initiated before they were referred.^7^

Pseudomonas Aeroginosa was the most common organism isolated in this study.This is probably because of its association with contact lenses.^23^,^24^,^25^,^26^ Staphylococcus and streptococcus were the next most frequent pathogens. Alsamarrai and Sunba from Kuwait have found that Staphyloccocus was the most frequent isolated pathogen. Streptococcus and Pseudomonas were also frequent isolated pathogens. Their study was performed in the late eighties when contact lenses were probably not widely used.^27^ Pseudomonas was the commonest organism cultured in Taiwan where the commonest risk factor was contact lens use.^10^ According to Basak and co-workers in their article of epidemiology of bacterial keratititis, streptococcus pneumonia was the predominant organism. Moreover, Streptococcus pneumonia was the commonest organism isolated in South of India as demonstrated by Baharathi and associates.^13^ Leck and associates have found that filamentous fungi are the commonest causative organism in microbial keratitis, and among the bacterial keratitis, streptococcus was the commonest.^22^ Staphylococcus was the commonest organism on a prospective clinical and microbiological study in Switzerland.^7^ It shows from the above the regional differences in the features of microbial keratitis especially in the risk factors and the causative organisms.

In this study, sleeping with the contact lenses was the most significant risk factor for contact lens related keratitis. This is consistent with other studies.^14^,^26^,^28^
